# The genome sequence of a nematode, *Thelazia callipaeda* Railliet & Henry, 1910 (Rhabditida: Thelaziidae)

**DOI:** 10.12688/wellcomeopenres.24942.2

**Published:** 2026-08-03

**Authors:** Ana Luísa Martins, Lewis Stevens, Manuela Kieninger, Wellcome Sanger Institute Tree of Life Management, Samples and Laboratory team, Wellcome Sanger Institute Scientific Operations: Sequencing Operations, Wellcome Sanger Institute Tree of Life Core Informatics team, Tree of Life Core Informatics collective, Joana Mourão, Irina Amorim, João Rodrigo Mesquita, Mark Blaxter

**Affiliations:** 1 School of Medicine and Biomedical Sciences (ICBAS), University of Porto, Porto, Portugal; 2 Tree of Life, Wellcome Sanger Institute, Hinxton, England, UK; 3 National Food Institute, Technical University of Denmark, Copenhagen, Denmark; 4 Instituto de Investigação e Inovação em Saúde (i3S), University of Porto, Porto, Portugal; 5 Associate Laboratory for Animal and Veterinary Sciences (AL4AnimalS), Lisbon, Portugal; 6 Instituto de Ciências, Tecnologias e Agroambiente (ICETA), University of Porto, Porto, Portugal

**Keywords:** Thelazia callipaeda; nematode; genome sequence; chromosomal; Rhabditida

## Abstract

We present a genome assembly of *Thelazia callipaeda* (nematode; Nematoda; Chromadorea; Rhabditida; Thelaziidae). The genome sequence has a total length of 117.59 megabases. Most of the assembly (75.82%) is scaffolded into 4 chromosomal pseudomolecules, including the X sex chromosome. The mitochondrial genome has been assembled, with a length of 13.66 kilobases.

## Species taxonomy

Eukaryota; Opisthokonta; Metazoa; Eumetazoa; Bilateria; Protostomia; Ecdysozoa; Nematoda; Chromadorea; Rhabditida; Spirurina; Spiruromorpha; Thelazioidea; Thelaziidae; *Thelazia*; *Thelazia callipaeda* Railliet & Henry, 1910 (NCBI: txid103827).

## Background

Thelaziosis is a zoonotic disease of growing concern, caused by nematodes of the genus *Thelazia* (Spirurida, Thelazioidea). Among these, *Thelazia callipaeda,* is a vector-borne nematode with zoonotic potential ( Lopes *et al.*, 2024; Sobotyk *et al.*, 2024). It affects a broad range of hosts ( do Vale *et al.*, 2019; Otranto & Traversa, 2005), including domestic animals such as dogs and cats ( Maia *et al.*, 2016; Pimenta *et al.*, 2013; Rodrigues *et al.*, 2012; Soares *et al.*, 2013; Vieira *et al.*, 2012) and various wild species from various families, such as Canidae, Felidae, Ursidae, Mustelidae, Procyonidae, Suidae and Leporidae ( do Vale *et al.*, 2020; Kitajima *et al.*, 2024; Otranto *et al.*, 2004; Otranto *et al.*, 2009; Papadopoulos *et al.*, 2022). This parasite may be a public health concern as it can also infect humans ( do Vale *et al.*, 2019; Hodžic *et al.*, 2014).

Parasitic nematodes of the family Thelaziidae infest the eyes of mammals and birds, contributing to significant veterinary and public health issues due to ocular disease ( Sobotyk *et al.*, 2024). The clinical manifestations of thelaziosis can vary widely in severity, from subclinical presentations to noticeable symptoms such as excessive tearing (epiphora), redness, increased ocular discharge, conjunctival edema, eye rubbing, light sensitivity (photophobia), conjunctivitis, keratitis, blepharospasm, purulent exudates, corneal ulcers, and even secondary glaucoma ( Marino *et al.*, 2021; Otranto & Traversa, 2005; Soares *et al.*, 2013). In severe cases, the condition may lead to blindness ( Otranto *et al.*, 2021). This eyeworm primarily infects the conjunctival sac, tear glands, tear ducts, and nictitating membrane of its definitive host, leading to various ophthalmological clinical signs ( do Vale *et al.*, 2020; Otranto *et al.*, 2021; Otranto & Traversa, 2005; Sobotyk *et al.*, 2024).

The parasite’s life cycle is indirect, involving both definitive hosts and intermediate vector hosts. The large drosophilid fruit fly *Phortica variegata* has been identified as the intermediate host for this nematode ( Marino *et al.*, 2018; Otranto *et al.*, 2006). The adult female *Thelazia callipaeda* releases first-stage larvae, which are ingested by the non-biting fly while feeding on the host’s lachrymal secretions ( Lopes *et al.*, 2024; Marino *et al.*, 2018; Otranto *et al.*, 2005; Otranto *et al.*, 2006; Otranto & Traversa, 2005). Transmission occurs when a male intermediate host ingests the first-stage larvae, which then molt into the infective third-stage larvae in the testes, migrate to the mouthparts, and are subsequently transmitted when the fly feeds on the lachrymal secretions of a new definitive host. The L3 larvae mature into adult worms, continuing the parasitic life cycle ( Otranto *et al.*, 2005; Otranto *et al.*, 2006; Otranto *et al.*, 2021).

Over the past decade, the geographic range and prevalence of *Thelazia callipaeda* infection have significantly expanded worldwide ( Otranto *et al.*, 2021; Sobotyk *et al.*, 2024). In Europe, Italy has reported the highest number of animal thelaziosis cases and was the first country to document the infection ( Otranto *et al.*, 2003; Otranto *et al.*, 2021). Over the last 30 years, there has been a gradual increase in the number of thelaziosis cases among a wide variety of hosts across Europe, with reported cases in France, Spain, Belgium, Switzerland, Portugal, Romania, the United Kingdom, Greece, Hungary, Austria, and Germany ( Caron *et al.*, 2013; do Vale *et al.*, 2019; Dorchies *et al.*, 2007; Farkas *et al.*, 2018; Graham-Brown *et al.*, 2017; Hodžic *et al.*, 2019; Ioniţə *et al.*, 2016; Marino *et al.*, 2021; Miró *et al.*, 2011; Miterpáková *et al.*, 2022; Motta *et al.*, 2014; Otranto *et al.*, 2009; Papadopoulos *et al.*, 2018; Sargo *et al.*, 2014; Silva *et al.*, 2020). Currently, the distribution of ocular thelaziosis continues to expand throughout Europe. Additionally, human cases have been reported over the past two decades in Italy, France, Serbia, Croatia, Germany, Spain, and Portugal ( Dolff *et al.*, 2020; Fuentes *et al.*, 2012; López Medrano *et al.*, 2015; Martínez-Sánchez *et al.*, 2021; Morgado *et al.*, 2022; Otranto & Dutto, 2008; Paradžik *et al.*, 2016; Tasić-Otašević *et al.*, 2016).

Approximately 45% of Europe’s human population, along with their domestic and companion animals, are at risk of this vector-borne zoonosis ( Masny *et al.*, 2013). Given the emergence of this zoonotic nematode in previously non-endemic regions and the need for more information regarding its ecology and epidemiology, the full impact of the disease on both dogs and humans remains underrecognized. Here, we present a chromosome-level reference genome for *Thelazia callipaeda* based on an adult female collected in Portugal.

## Methods

### Sample acquisition

The *Thelazia callipaeda* specimen used for genome sequencing (specimen ID SAN20002664, ToLID nxTheCall6) was collected from the eye of a domestic dog in Porto, Portugal (latitude 41.1476, longitude −8.6177) on 2023-09-02. The specimen was collected and identified by Ana Luísa Martins (Instituto De Ciências Biomédicas Abel Salazar). A second specimen collected from the same host on the same occasion was used for Hi-C sequencing (specimen ID SAN20002659, ToLID nxTheCall1). Sample metadata were collected in line with the best practice guidelines for the Earth Biogenome Project described by Lawniczak *et al*. (2025).

### Nucleic acid extraction

High molecular weight DNA was extracted from a frozen adult of *Thelazia callipaeda* (nxTheCall6). DNA extraction was performed using the Monarch HMW DNA Extraction Kit for Tissue (NEB) with the following modifications. A 50 μl aliquot of cold lysis buffer mix containing Proteinase K was added to the frozen worm. The sample was pestled in a 1.5 ml pestle tube for approximately 1 minute. Subsequently, 550 μl of lysis buffer mix was added, and the mixture was gently inverted several times to mix.

The sample was then incubated in a Thermomixer C (Eppendorf ) at 56 °C and 750 rpm. After 15 minutes, the sample was removed, and the remaining worm tissue was allowed to settle. The tissue was then further disintegrated by additional pestling until fully broken down. The sample was returned to the Thermomixer C and digested for two hours at 56 °C and at 550 rpm. RNase A (10 μl) was added 30 minutes before the end of the digestion.

Following digestion, the sample was centrifuged for 5 minutes at 2500 *g.* The resulting supernatant was transferred to a new 1.5 ml LoBind microtube (Eppendorf ) and incubated on ice for 5 minutes. Cold protein separation solution (300 μl, Monarch Kit) was added, and the sample was inverted for 1 minute before being returned to ice for 10 minutes. The sample was then centrifuged at 16 000 *g* for 25 minutes at room temperature, and the supernatant was transferred to a new 2 ml tube.

Isopropanol precipitation was carried out on the transferred supernatant. DNA was eluted in a total volume of 200 μl elution buffer. The HMW DNA was sheared using the Megaruptor 3 (Diagenode) with a setting of 36. The sheared DNA was cleaned using 0.81× of the ProNex® Size-Selective Purification System (Promega) via SPRI bead purification.

The concentration of the sheared and purified DNA was assessed using a Nanodrop spectrophotometer and Qubit Fluorometer using the Qubit dsDNA High Sensitivity Assay kit. Fragment size distribution was evaluated by running the sample on the FemtoPulse system.

### PacBio HiFi library preparation and sequencing

Library preparation and sequencing were performed at the WSI Scientific Operations core. Prior to library preparation, the DNA was fragmented to ~10 kb. Ultra-low-input (ULI) libraries were prepared using the PacBio SMRTbell® Express Template Prep Kit 2.0 and gDNA Sample Amplification Kit. Samples were normalised to 20 ng DNA. Single-strand overhang removal, DNA damage repair, and end-repair/A-tailing were performed according to the manufacturer’s instructions, followed by adapter ligation. A 0.85× pre-PCR clean-up was carried out with Promega ProNex beads.

The DNA was evenly divided into two aliquots for dual PCR (reactions A and B), both following the manufacturer’s protocol. A 0.85× post-PCR clean-up was performed with ProNex beads. DNA concentration was measured using a Qubit Fluorometer v4.0 (Thermo Fisher Scientific) with the Qubit HS Assay Kit, and fragment size was assessed on an Agilent Femto Pulse Automated Pulsed Field CE Instrument (Agilent Technologies) using the gDNA 55 kb BAC analysis kit. PCR reactions A and B were then pooled, ensuring a total mass of ≥500 ng in 47.4 μl.

The pooled sample underwent another round of DNA damage repair, end-repair/A-tailing, and hairpin adapter ligation. A 1× clean-up was performed with ProNex beads, followed by DNA quantification using the Qubit and fragment size analysis using the Agilent Femto Pulse. Size selection was performed on the Sage Sciences PippinHT system, with target fragment size determined by Femto Pulse analysis (typically 4–9 kb). Size-selected libraries were cleaned with 1.0× ProNex beads and normalised to 2 nM before sequencing.

The sample was sequenced on a Revio instrument (Pacific Biosciences). The prepared library was normalised to 2 nM, and 15 μL was used for making complexes. Primers were annealed and polymerases bound to generate circularised complexes, following the manufacturer’s instructions. Complexes were purified using 1.2X SMRTbell beads, then diluted to the Revio loading concentration (200–300 pM) and spiked with a Revio sequencing internal control. The sample was sequenced on a Revio 25 M SMRT cell. The SMRT Link software (Pacific Biosciences), a web-based workflow manager, was used to configure and monitor the run and to carry out primary and secondary data analysis.

### Hi-C

***Sample preparation and crosslinking***

Hi-C data were generated from the whole organism tissue of nxTheCall1 using the Arima-HiC v2 kit (Arima Genomics). Tissue was finely ground using the Covaris cryoPREP Dry Pulverizer (Covaris), and then subjected to nuclei isolation. Nuclei were isolated using a modified protocol based on the Qiagen QProteome Cell Compartment Kit (Qiagen), in which only the Lysis and CE2 buffers were used, with QIAshredder spin columns. After isolation, nuclei were fixed using formaldehyde to a final concentration of 2% to crosslink the DNA. The crosslinked DNA was then digested and biotinylated according to the manufacturer’s instructions. A clean-up step was performed with SPRIselect beads before library preparation. DNA concentration was quantified using the Qubit Fluorometer v4.0 (Thermo Fisher Scientific) and the Qubit HS Assay Kit, following the manufacturer’s instructions.

***Hi-C library preparation and sequencing***

Biotinylated DNA constructs were fragmented using a Covaris E220 sonicator and size selected to 400–600 bp using SPRISelect beads. DNA was enriched with Arima-HiC v2 kit Enrichment beads. End repair, A-tailing, and adapter ligation were carried out with the NEBNext Ultra II DNA Library Prep Kit (New England Biolabs), following a modified protocol where library preparation occurs while DNA remains bound to the Enrichment beads. Library amplification was performed using KAPA HiFi HotStart mix and a custom Unique Dual Index (UDI) barcode set (Integrated DNA Technologies). Depending on sample concentration and biotinylation percentage determined at the crosslinking stage, libraries were amplified with 10–16 PCR cycles. Post-PCR clean-up was performed with SPRISelect beads. Libraries were quantified using the AccuClear Ultra High Sensitivity dsDNA Standards Assay Kit (Biotium) and a FLUOstar Omega plate reader (BMG Labtech).

Prior to sequencing, libraries were normalised to 10 ng/μL. Normalised libraries were quantified again and equimolar and/or weighted 2.8 nM pools were created. Pool concentrations were checked using the Agilent 4200 TapeStation (Agilent) with High Sensitivity D500 reagents before sequencing. Sequencing was performed using paired-end 150 bp reads on the Illumina NovaSeq X.

### Genome assembly

Prior to assembly of the PacBio HiFi reads, a database of *k*-mer counts ( *k* = 31) was generated from the filtered reads using FastK. GenomeScope2 ( Ranallo-Benavidez *et al.*, 2020) was used to analyse the *k*-mer frequency distributions, providing estimates of genome size, heterozygosity, and repeat content.

The HiFi reads were assembled using Hifiasm ( Cheng *et al.*, 2021) with the --primary option. The Hi-C reads ( Rao *et al.*, 2014) were mapped to the primary contigs using bwa-mem2 ( Vasimuddin *et al.*, 2019), and the contigs were scaffolded in YaHS ( Zhou *et al.*, 2023) with the --break option for handling potential misassemblies. The scaffolded assemblies were evaluated using Gfastats ( Formenti *et al.*, 2022), BUSCO ( Manni *et al.*, 2021) and MERQURY.FK ( Rhie *et al.*, 2020). The mitochondrial genome was assembled using MitoHiFi ( Uliano-Silva *et al.*, 2023).

### Assembly curation

The assembly was decontaminated using the Assembly Screen for Cobionts and Contaminants (ASCC) pipeline. TreeVal was used to generate the flat files and maps for use in curation. Manual curation was conducted primarily in PretextView and HiGlass ( Kerpedjiev *et al.*, 2018). Scaffolds were visually inspected and corrected as described by Howe *et al*. (2021). Manual corrections included 11 breaks and 36 joins. The X chromosome was identified by read coverage. The curation process is documented at https://gitlab.com/wtsi-grit/rapid-curation.PretextSnapshot was used to generate a Hi-C contact map of the final assembly.

### Assembly analysis and quality assessment

The Merqury.FK tool ( Rhie *et al.*, 2020) was used to evaluate *k*-mer completeness and assembly quality for the primary and alternate haplotypes using the *k*-mer databases ( *k* = 31) computed prior to genome assembly. The analysis outputs included assembly QV scores and completeness statistics.

The genome was analysed using the BlobToolKit pipeline, a Nextflow implementation of the earlier Snakemake version ( Challis *et al.*, 2020). PacBio reads were aligned to the assembly using minimap2 ( Li, 2018) and processed with SAMtools ( Danecek *et al.*, 2021) to generate coverage tracks. It runs BUSCO ( Manni *et al.*, 2021) using lineages identified from NCBI Taxonomy. For the three domain-level lineages, BUSCO genes are aligned to the UniProt Reference Proteomes database ( Bateman *et al.*, 2023) using DIAMOND blastp ( Buchfink *et al.*, 2021). The genome is divided into chunks based on the density of BUSCO genes from the closest taxonomic lineage, and each chunk is aligned to the UniProt Reference Proteomes database with DIAMOND blastx. Sequences without hits are chunked using seqtk and aligned to the NCBI NT database with blastn ( Altschul *et al.*, 1990). The BlobToolKit suite consolidates all outputs into a blobdir for visualisation. The BlobToolKit pipeline was developed using nf-core tooling ( Ewels *et al.*, 2020) and MultiQC ( Ewels *et al.*, 2016), with package management via Conda and Bioconda ( Grüning *et al.*, 2018), and containerisation through Docker ( Merkel, 2014) and Singularity ( Kurtzer *et al.*, 2017).

The distribution of Nigon elements, which represent the seven ancestral linkage groups in rhabditid nematodes ( Gonzalez de la Rosa *et al.*, 2021), was visualised by providing the ‘full_table.tsv’ file from the BUSCO nematoda_odb10 output to the R script vis_ALG.R (available at https://github.com/lstevens17/vis_ALG_gn ), using a window size of 500 kb.

## Genome sequence report

### Sequence data

**Figure 1.**
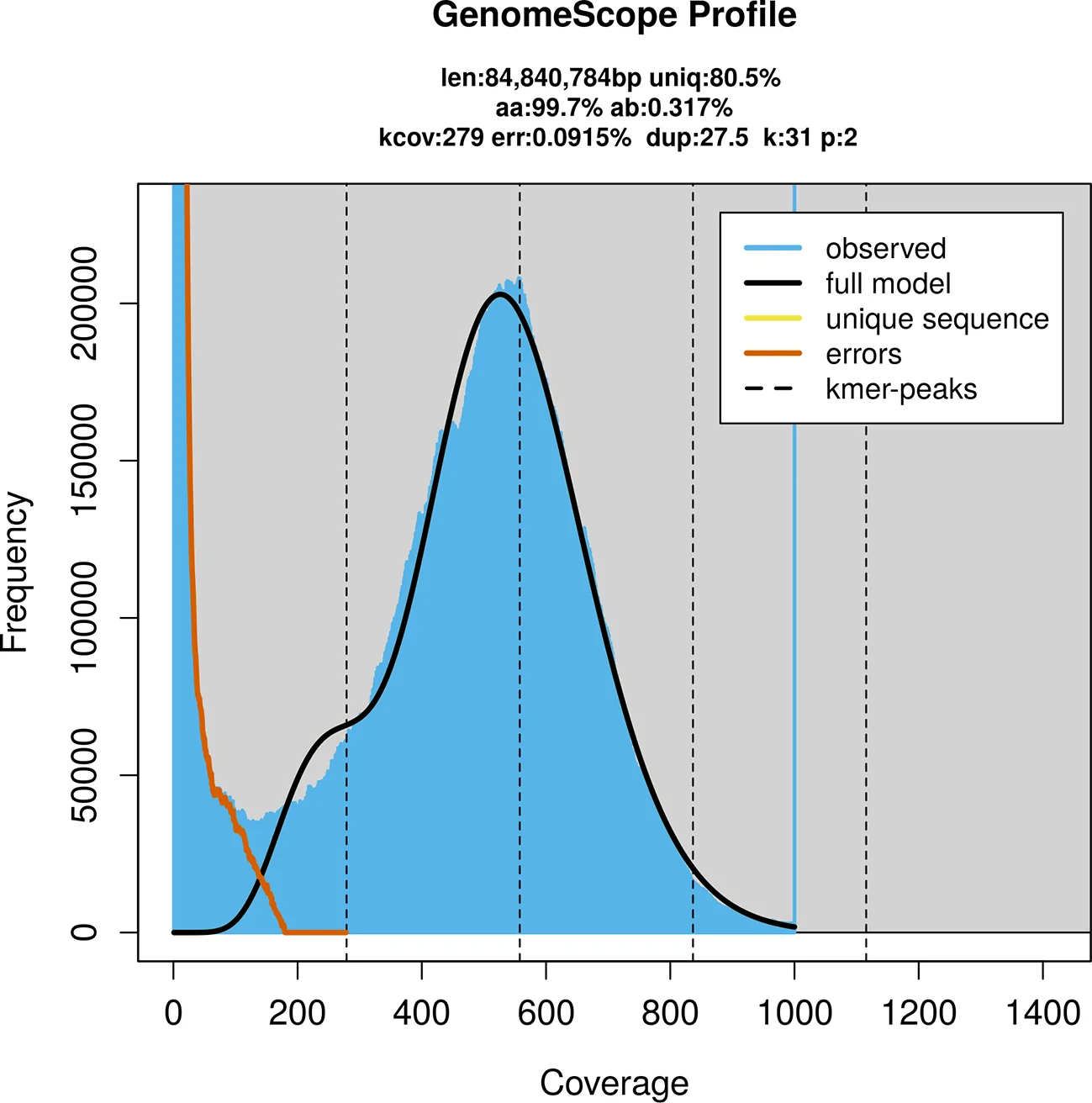
Frequency distribution of *k*-mers generated using GenomeScope2. The plot shows observed and modelled *k*-mer spectra, providing estimates of genome size, heterozygosity, and repeat content based on unassembled sequencing reads.

The genome of a specimen of *Thelazia callipaeda* was sequenced using Pacific Biosciences single-molecule HiFi long reads, generating 51.21 Gb (gigabases) from 4.46 million reads, which were used to assemble the genome. GenomeScope2.0 analysis estimated the haploid genome size at 84.84 Mb, with a heterozygosity of 0.32% and repeat content of 19.63% ( Figure 1). These estimates guided expectations for the assembly. Based on the estimated genome size, the sequencing data provided approximately 558× coverage. Hi-C sequencing produced 134.05 Gb from 887.72 million reads, which were used to scaffold the assembly. Table 1 summarises the specimen and sequencing details.

**Table 1.** Specimen and sequencing data for BioProject PRJEB83517.

Platform	PacBio HiFi	Hi-C
**ToLID**	nxTheCall6	nxTheCall1
**Specimen ID**	SAN20002664	SAN20002659
**BioSample (source individual)**	SAMEA115373893	SAMEA115373888
**BioSample (tissue)**	SAMEA115373921	SAMEA115373916
**Tissue**	whole organism	whole organism
**Instrument**	Revio	Illumina NovaSeq X
**Run accessions**	ERR14106129	ERR14075554
**Read count total**	4.46 million	887.72 million
**Base count total**	51.21 Gb	134.05 Gb

### Assembly statistics

The primary haplotype was assembled, and contigs corresponding to an alternate haplotype were also deposited in INSDC databases. The final assembly has a total length of 117.59 Mb in 104 scaffolds, with 121 gaps, and a scaffold N50 of 15.22 Mb ( Table 2).

**Table 2.** Genome assembly statistics.

Assembly name	nxTheCall6.1
**Assembly accession**	GCA_965194785.1
**Alternate haplotype accession**	GCA_965194775.1
**Assembly level**	chromosome
**Span (Mb)**	117.59
**Number of chromosomes**	4
**Number of contigs**	225
**Contig N50**	1.01 Mb
**Number of scaffolds**	104
**Scaffold N50**	15.22 Mb
**Organelles**	Mitochondrion: 13.66 kb

**Figure 2.**
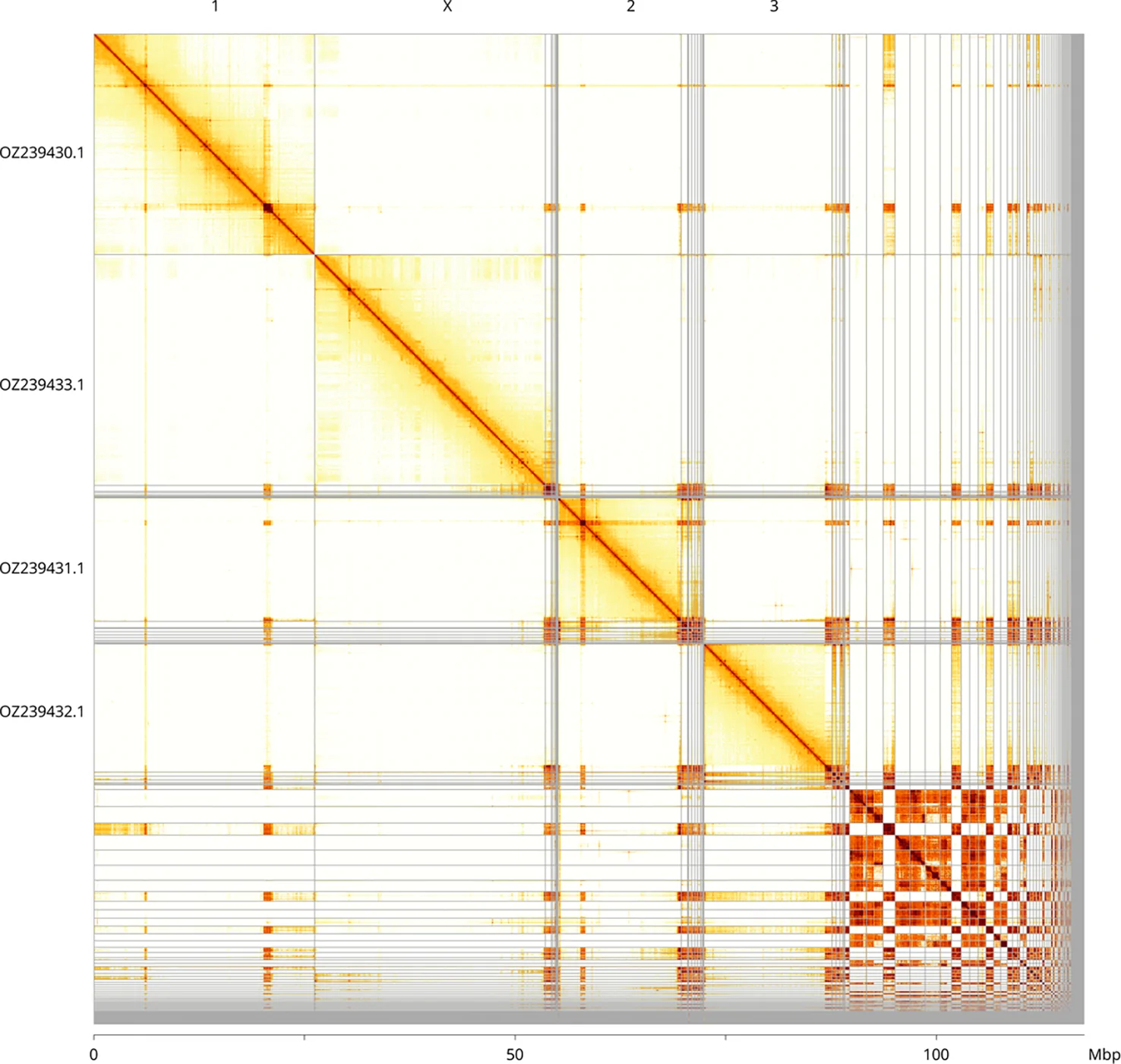
Hi-C contact map of the *Thelazia callipaeda* genome assembly.

Most of the assembly sequence (75.82%) was assigned to 4 chromosomal-level scaffolds, representing 3 autosomes and the X sex chromosome. These chromosome-level scaffolds, confirmed by Hi-C data, are named according to size ( Figure 2; Table 3). The unplaced scaffolds may represent fragments of a highly degenerate Y chromosome, as the HiFi data originated from a mated female (containing male-derived reads). It is also possible that they represent B chromosome fragments.

Assembled chromosomes are shown in order of size and labelled along the axes. The plot was generated using PretextSnapshot.

**Table 3.** Chromosomal pseudomolecules in the primary genome assembly of *Thelazia callipaeda* nxTheCall6.

INSDC accession	Molecule	Length (Mb)	GC%
OZ239430.1	1	28.89	30.50
OZ239431.1	2	17.32	29
OZ239432.1	3	16.73	29.50
OZ239433.1	X	26.22	30.50

The mitochondrial genome was also assembled. This sequence is included as a contig in the multifasta file of the genome submission and as a standalone record.

**Figure 3.**
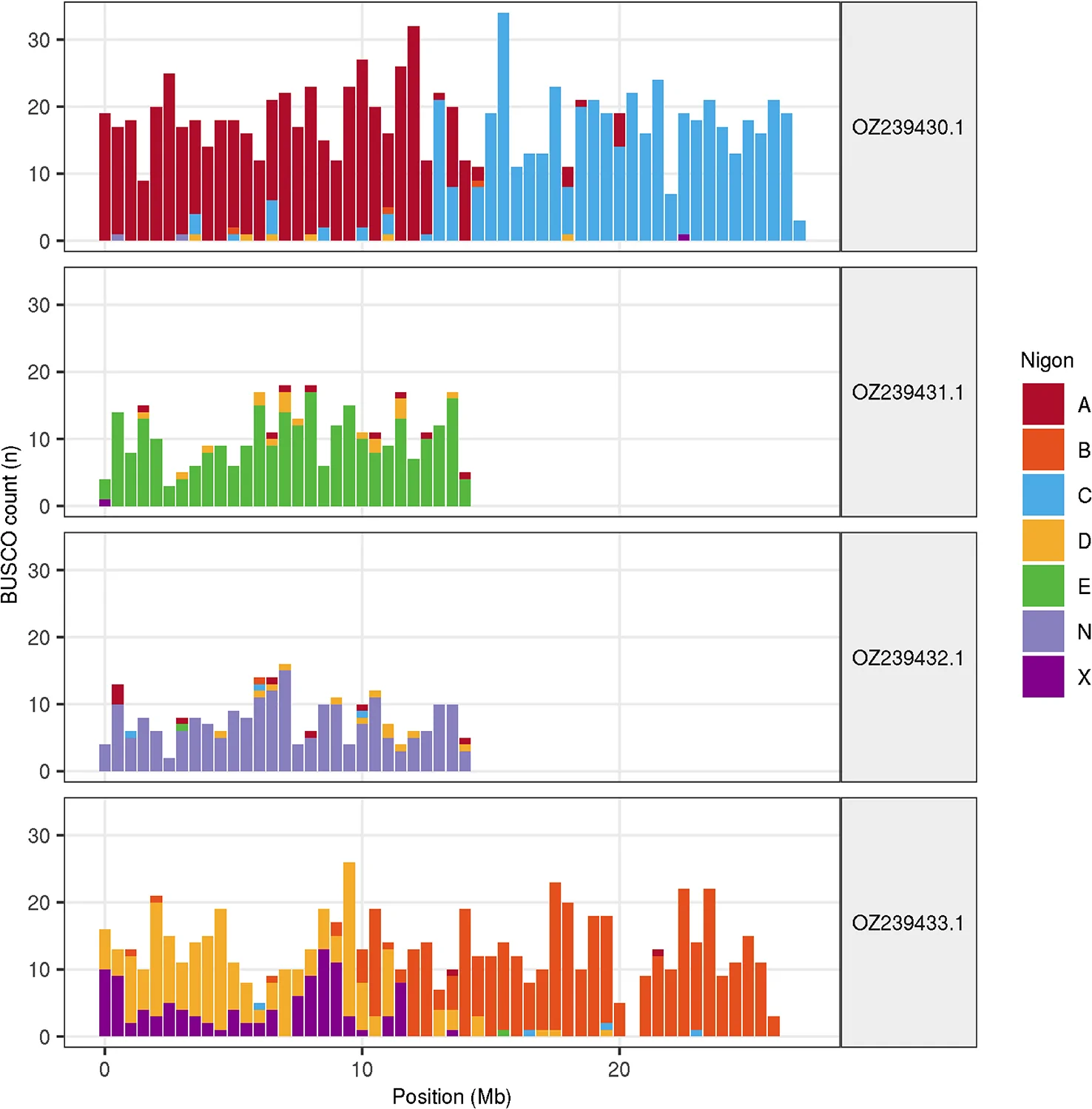
Nigon element painting of chromosomes in the nxTheCall6.1 assembly of *Thelazia callipaeda.* Counts of BUSCO loci in 500 kb windows are coloured by their allocation to the seven Nigon elements (A-E, N, X).

Chromosome painting with Nigon elements illustrates the distribution of orthologues along chromosomes and highlights patterns of chromosomal evolution relative to ancestral linkage groups ( Figure 3).

### Assembly quality metrics

**Figure 4.**
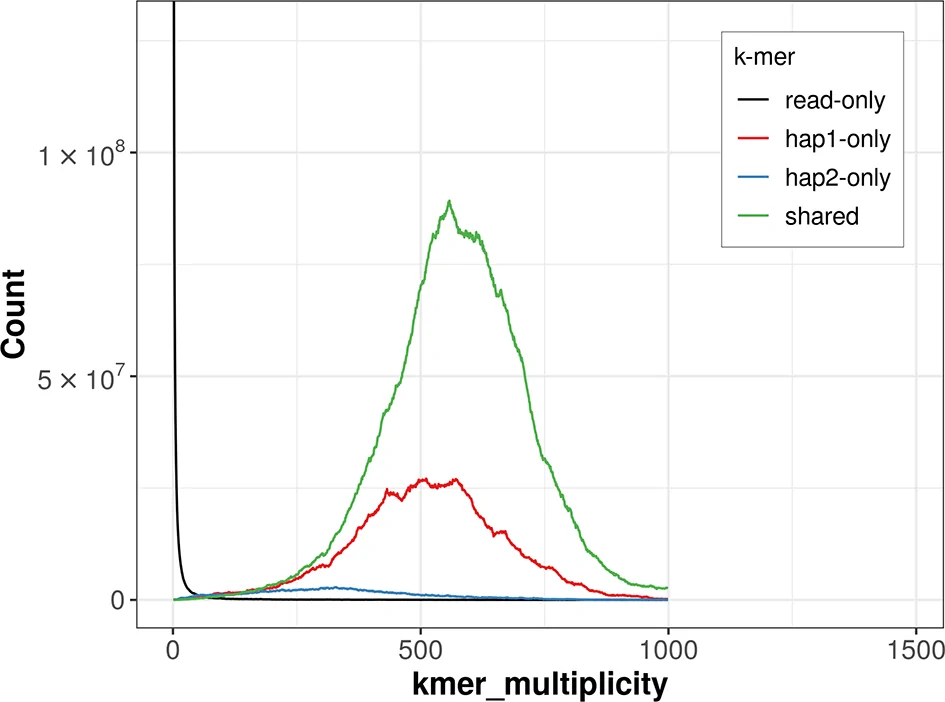
Evaluation of *k*-mer completeness using MerquryFK. This plot illustrates the recovery of *k*-mers from the original read data in the final assemblies. The horizontal axis represents *k*-mer multiplicity, and the vertical axis shows the number of *k*-mers. The black curve represents *k*-mers that appear in the reads but are not assembled. The green curve corresponds to *k*-mers shared by both haplotypes, and the red and blue curves show *k*-mers found only in one of the haplotypes.

The combined primary and alternate assemblies achieve an estimated QV of 58.5. The *k*-mer completeness is 93.47% for the primary assembly, 72.22% for the alternate haplotype, and 98.84% for the combined assemblies ( Figure 4).

**Figure 5.**
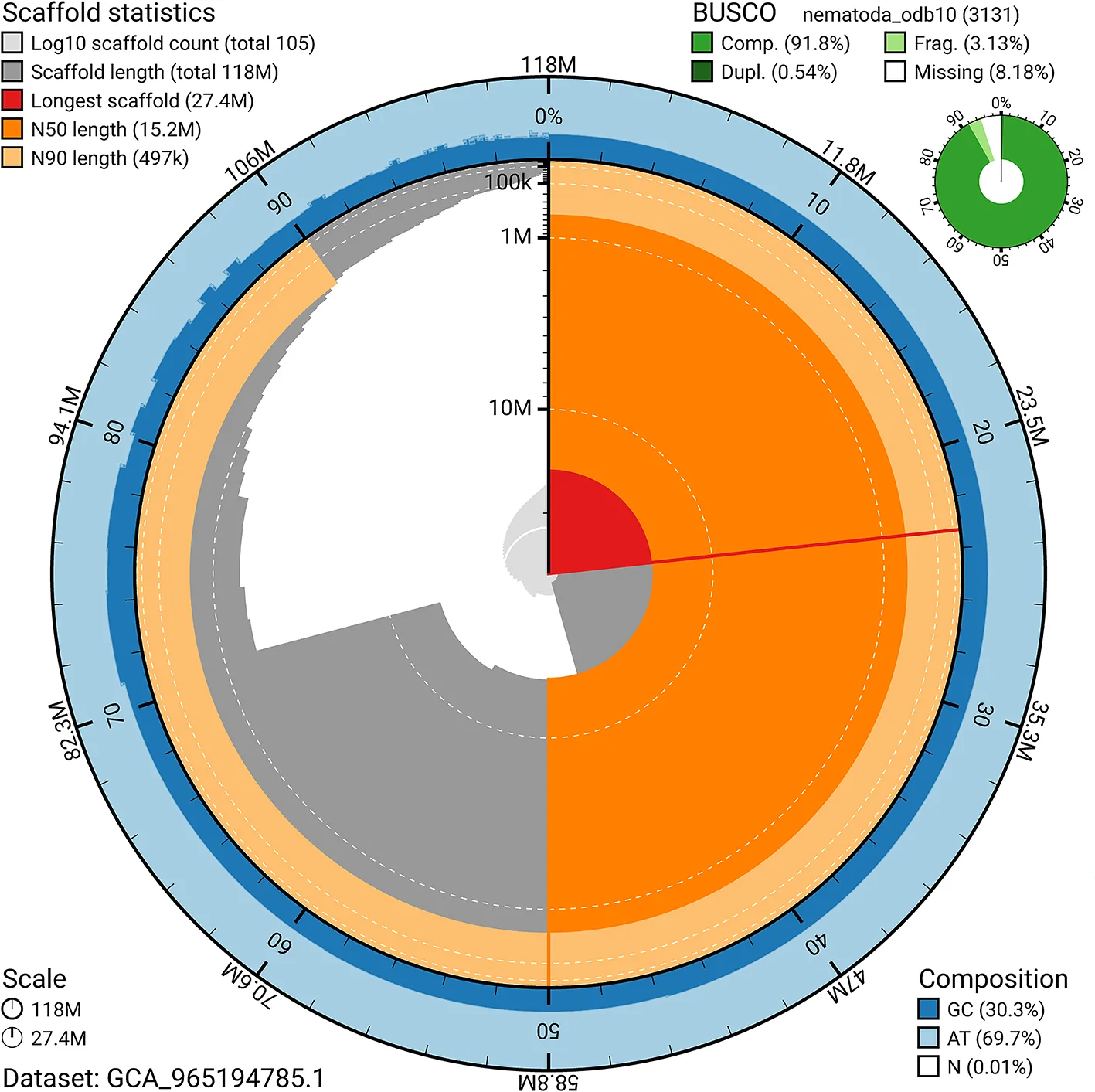
Assembly metrics for nxTheCall6.1.

**Figure 6.**
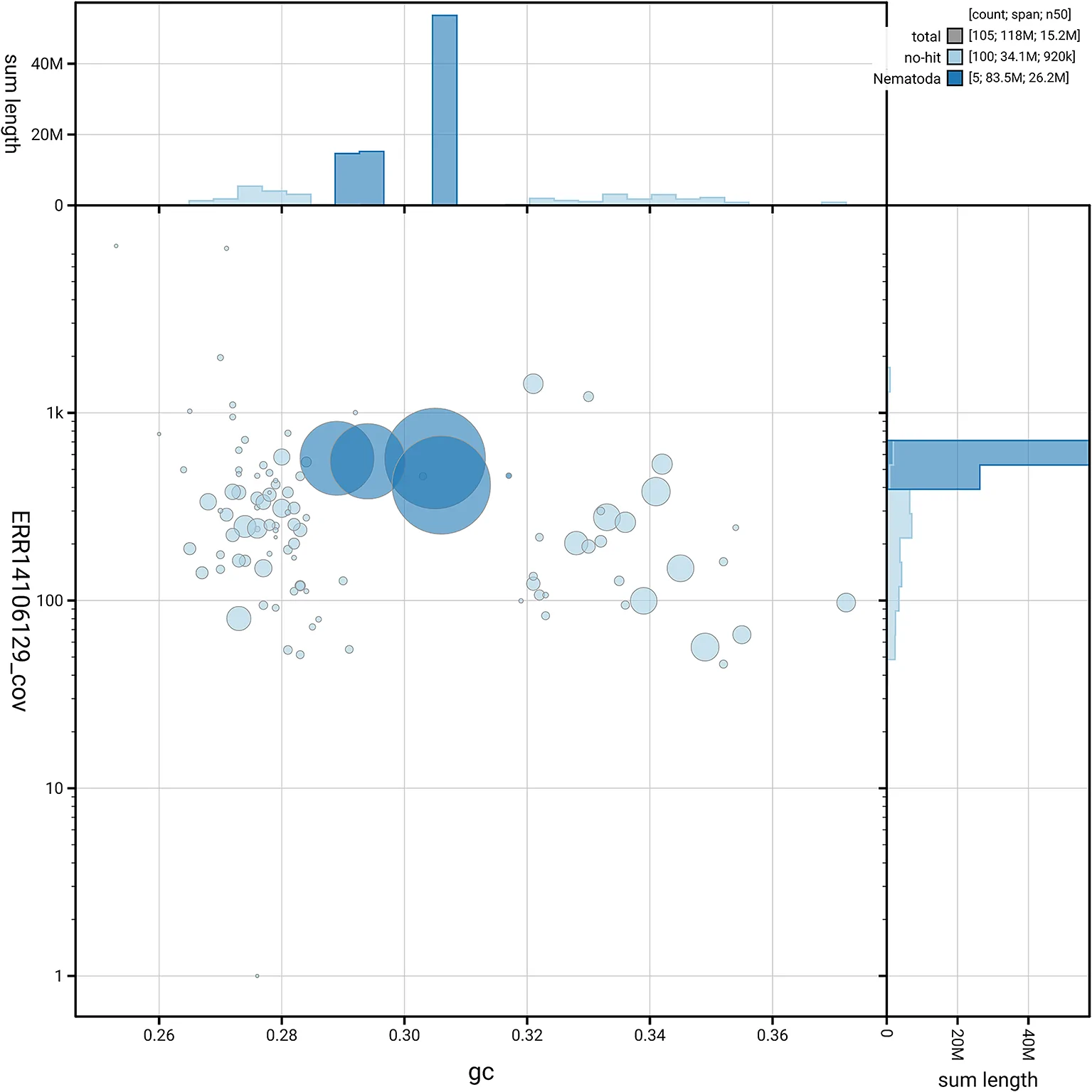
BlobToolKit GC-coverage plot for nxTheCall6.1. Blob plot showing sequence coverage (vertical axis) and GC content (horizontal axis). The circles represent scaffolds, with the size proportional to scaffold length and the colour representing phylum membership. The histograms along the axes display the total length of sequences distributed across different levels of coverage and GC content. An interactive version of this figure is available on the BlobToolKit viewer.

BUSCO v.5.7.1 analysis using the nematoda_odb10 reference set [ *n* = 3 131; Kriventseva *et al.* (2019)] identified 91.8% of the expected gene set (single = 91.3%, duplicated = 0.5%). The snail plot in Figure 5 summarises the scaffold length distribution and other assembly statistics for the primary assembly. The blob plot in Figure 6 shows the distribution of scaffolds by GC proportion and coverage.

The BlobToolKit snail plot provides an overview of assembly metrics and BUSCO gene completeness. The circumference represents the length of the whole genome sequence, and the main plot is divided into 1,000 bins around the circumference. The outermost blue tracks display the distribution of GC, AT, and N percentages across the bins. Scaffolds are arranged clockwise from longest to shortest and are depicted in dark grey. The longest scaffold is indicated by the red arc, and the deeper orange and pale orange arcs represent the N50 and N90 lengths. A light grey spiral at the centre shows the cumulative scaffold count on a logarithmic scale. A summary of complete, fragmented, duplicated, and missing BUSCO genes in the nematoda_odb10 set is presented at the top right. An interactive version of this figure can be accessed on the BlobToolKit viewer.

Table 4 lists the assembly metric benchmarks adapted from Rhie *et al.* (2021) the Earth BioGenome Project Report on Assembly Standards September 2024. The EBP metric calculated for the primary assembly is **6.7.Q62**.

**Table 4.** Earth Biogenome Project summary metrics for the *Thelazia callipaeda* assembly.

Measure	Value	Benchmark
EBP summary (primary)	6.7.Q62	5.C.Q40 *
Contig N50 length	1.01 Mb	≥ 1 Mb
Scaffold N50 length	15.22 Mb	= chromosome N50
Consensus quality (QV)	Primary: 62.7; alternate: 57.3; combined: 58.5	≥ 40
*k*-mer completeness	Primary: 93.47%; alternate: 72.22%; combined: 98.84%	≥ 90%
BUSCO	C:91.8% [S:91.3%; D:0.5%]; F:3.1%; M:5.0%; n:3 131	S > 90%; D < 5%
Percentage of assembly assigned to chromosomes	75.82%	≥ 90%

* Standard recommended for species with limited material per individual.

### Wellcome Sanger Institute – Legal and Governance

The materials that have contributed to this genome note have been supplied by a Tree of Life collaborator. The Wellcome Sanger Institute employs a process whereby due diligence is carried out proportionate to the nature of the materials themselves, and the circumstances under which they have been/are to be collected and provided for use. The purpose of this is to address and mitigate any potential legal and/or ethical implications of receipt and use of the materials as part of the research project, and to ensure that in doing so, we align with best practice wherever possible. The overarching areas of consideration are:

• Ethical review of provenance and sourcing of the material.
• Legality of collection, transfer and use (national and international).

Each transfer of samples is undertaken according to a Research Collaboration Agreement or Material Transfer Agreement entered into by the Tree of Life collaborator, Genome Research Limited (operating as the Wellcome Sanger Institute), and in some circumstances, other Tree of Life collaborators.

## Author information

Members of the Wellcome Sanger Institute Tree of Life Management, Samples and Laboratory team

Members of Wellcome Sanger Institute Scientific Operations – Sequencing Operations

Members of the Wellcome Sanger Institute Tree of Life Core Informatics team

Members of the Tree of Life Core Informatics collective

## Data Availability

European Nucleotide Archive: Thelazia callipaeda (thelaziosis nematode). Accession number PRJEB83517. The genome sequence is released openly for reuse. The *Thelazia callipaeda* genome sequencing initiative is part of the Sanger Institute Tree of Life Programme (PRJEB43745) and 959NG (PRJEB81973). All raw sequence data and the assembly have been deposited in INSDC databases. The genome will be annotated using available RNA-Seq data and presented through the Ensembl pipeline at the European Bioinformatics Institute. Raw data and assembly accession identifiers are reported in Table 1 and Table 2. Pipelines used for genome assembly at the WSI Tree of Life are available at https://pipelines.tol.sanger.ac.uk/pipelines. Table 5 lists software versions used in this study.

## References

[ref1] Altschul SF Gish W Miller W : Basic Local Alignment Search Tool. *J Mol Biol.* 1990;215(3):403–410. 10.1016/S0022-2836(05)80360-2 2231712

[ref2] Bateman A Martin MJ Orchard S : Uni Prot: the Universal Protein Knowledgebase in 2023. *Nucleic Acids Res.* 2023;51(D1):D523–D531. 10.1093/nar/gkac1052 36408920 PMC9825514

[ref3] Buchfink B Reuter K : Drost HG: Sensitive protein alignments at Tree-of-Life scale using DIAMOND. *Nat Methods.* 2021;18(4):366–368. 10.1038/s41592-021-01101-x 33828273 PMC8026399

[ref4] Caron Y Premont J Losson B : *Thelazia callipaeda* ocular infection in two dogs in Belgium. *J Small Anim Pract.* 2013;54(4):205–208. 10.1111/jsap.12003 23278915

[ref5] Challis R Richards E Rajan J : BlobToolKit - Interactive Quality Assessment of Genome Assemblies. *G3 (Bethesda).* 2020;10(4):1361–1374. 10.1534/g3.119.400908 32071071 PMC7144090

[ref6] Cheng H Concepcion GT Feng X : Haplotype-resolved *de novo* assembly using phased assembly graphs with hifiasm. *Nat Methods.* 2021;18(2):170–175. 10.1038/s41592-020-01056-5 33526886 PMC7961889

[ref7] Danecek P Bonfield JK Liddle J : Twelve years of SAMtools and BCFtools. *Giga Science.* 2021;10(2):giab 008. 10.1093/gigascience/giab008 33590861 PMC7931819

[ref8] Vale B do Lopes AP Conceição Fontes M da : Thelaziosis due to *Thelazia callipaeda* in Europe in the 21st century - a review. *Vet Parasitol.* 2019;275:108957. 10.1016/j.vetpar.2019.108957 31630050

[ref9] Vale B do Lopes AP Conceição Fontes M da : Systematic review on infection and disease caused by *Thelazia callipaeda* in Europe: 2001–2020. *Parasite.* 2020;27:52. 10.1051/parasite/2020048 32996882 PMC7526429

[ref10] Dolff S Kehrmann J Eisermann P : Case report: *Thelazia callipaeda* eye infection: the first human case in Germany. *Am J Trop Med Hyg.* 2020;102(2):350–351. 10.4269/ajtmh.19-0483 31912777 PMC7008349

[ref11] Dorchies P Chaudieu G Siméon LA : First reports of autochthonous eyeworm infection by *Thelazia callipaeda* (Spirurida, Thelaziidae) in dogs and cat from France. *Vet Parasitol.* 2007;149(3–4):294–297. 10.1016/j.vetpar.2007.08.005 17854998

[ref12] Ewels P Magnusson M Lundin S : Multi QC: summarize analysis results for multiple tools and samples in a single report. *Bioinformatics.* 2016;32(19):3047–3048. 10.1093/bioinformatics/btw354 27312411 PMC5039924

[ref13] Ewels PA Peltzer A Fillinger S : The nf-core framework for community-curated bioinformatics pipelines. *Nat Biotechnol.* 2020;38(3):276–278. 10.1038/s41587-020-0439-x 32055031

[ref14] Farkas R Takács N Gyurkovszky M : The first feline and new canine cases of *Thelazia callipaeda* (Spirurida: Thelaziidae) infection in Hungary. *Parasit Vectors.* 2018;11(1):338. 10.1186/s13071-018-2925-2 29884211 PMC5993998

[ref15] Formenti G Abueg L Brajuka A : Gfastats: conversion, evaluation and manipulation of genome sequences using assembly graphs. *Bioinformatics.* 2022;38(17):4214–4216. 10.1093/bioinformatics/btac460 35799367 PMC9438950

[ref16] Fuentes I Montes I Saugar JM : Thelaziosis in humans, a zoonotic infection, Spain, 2011. *Emerg Infect Dis.* 2012;18(12):2073–2075. 10.3201/eid1812.1204723557880 23182166 PMC3557880

[ref17] Gonzalez de la Rosa PM Thomson M Trivedi U : A telomere-to-telomere assembly of Oscheius tipulaeand the evolution of rhabditid nematode chromosomes. *G3 (Bethesda).* 2021;11(1):jkaa 020. 10.1093/g3journal/jkaa020 33561231 PMC8022731

[ref18] Graham-Brown J Gilmore P Colella V : Three cases of imported eyeworm infection in dogs: a new threat for the United Kingdom. *Vet Rec.* 2017;181(13):346. 10.1136/vr.104378 28870975 PMC5738592

[ref19] Grüning B Dale R Sjödin A : Bioconda: sustainable and comprehensive software distribution for the life sciences. *Nat Methods.* 2018;15(7):475–476. 10.1038/s41592-018-0046-7 29967506 PMC11070151

[ref20] Hodžić A Latrofa MS Annoscia G : The spread of zoonotic *Thelazia callipaeda* in the Balkan area. *Parasit Vectors.* 2014;7:352. 10.1186/1756-3305-7-352 25078286 PMC4124150

[ref21] Hodžić A Payer A Duscher GG : The first autochthonous case of feline ocular thelaziosis in Austria. *Parasitol Res.* 2019;118(4):1321–1324. 10.1007/s00436-019-06275-0 30826924 PMC6426986

[ref22] Howe K Chow W Collins J : Significantly improving the quality of genome assemblies through curation. *Giga Sci.* 2021;10(1):giaa 153. 10.1093/gigascience/giaa153 33420778 PMC7794651

[ref23] Ioniţă M Mitrea IL Ionică AM : New cases of *Thelazia callipaeda* Haplotype 1 in dogs suggest a wider distribution in Romania. *Vector Borne Zoonotic Dis.* 2016;16(3):172–175. 10.1089/vbz.2015.1919 26854520

[ref24] Kerpedjiev P Abdennur N Lekschas F : HiGlass: web-based visual exploration and analysis of genome interaction maps. *Genome Biol.* 2018;19(1):125. 10.1186/s13059-018-1486-1 30143029 PMC6109259

[ref25] Kitajima A Tokiwa T Doi K : New host record of *Thelazia callipaeda* (Nematoda: Spirurida) with a notably wide host range and shared zoonotic lineage in Japan. *Parasitol Int.* 2024;102:102913. 10.1016/j.parint.2024.102913 38885786

[ref26] Kriventseva EV Kuznetsov D Tegenfeldt F : Ortho DB v10: sampling the diversity of animal, plant, fungal, protist, bacterial and viral genomes for evolutionary and functional annotations of orthologs. *Nucleic Acids Res.* 2019;47(D1):D807–D811. 10.1093/nar/gky1053 30395283 PMC6323947

[ref27] Kurtzer GM Sochat V Bauer MW : Singularity: scientific containers for mobility of compute. *PLoS One.* 2017;12(5):e0177459. 10.1371/journal.pone.0177459 28494014 PMC5426675

[ref28] Lawniczak MKN Kocot KM Astrin JJ : Best-practice guidance for Earth Bio Genome Project sample collection and processing: Progress and challenges in biodiverse reference genome creation *Giga Science.* 2025;14:giaf 041. , 10.1093/gigascience/giaf041 40440092 PMC12121479

[ref29] Li H : Minimap 2: pairwise alignment for nucleotide sequences. *Bioinformatics.* 2018;34(18):3094–3100. 10.1093/bioinformatics/bty191 29750242 PMC6137996

[ref30] Lopes AF Ferreira MR Vale B do : Update on infections with *Thelazia callipaeda* in European wildlife and a report in a red fox, *Vulpes vulpes*, in Portugal. *Curr Res Parasitol Vector Borne Dis.* 2024;6:100211. 10.1016/j.crpvbd.2024.100211 39280995 PMC11399657

[ref31] López Medrano R Guerra Calleja G Díez Morrondo C : Ocular thelaziosis, an emergent zoonosis in Spain. *Med Clin (Barc).* 2015;145(7):317–318. 10.1016/j.medcli.2015.02.014 25851914

[ref32] Maia C Catarino AL Almeida B : Emergence of *Thelazia callipaeda* infection in dogs and cats from east-central Portugal. *Transbound Emerg Dis.* 2016;63(4):416–421. 10.1111/tbed.12284 25382165

[ref33] Manni M Berkeley MR Seppey M : BUSCO update: novel and streamlined workflows along with broader and deeper phylogenetic coverage for scoring of eukaryotic, prokaryotic, and viral genomes. *Mol Biol Evol.* 2021;38(10):4647–4654. 10.1093/molbev/msab199 34320186 PMC8476166

[ref34] Marino V Gálvez R Colella V : Detection of *Thelazia callipaeda* in *Phortica variegata* and spread of canine thelaziosis to new areas in Spain. *Parasit Vectors.* 2018;11(1):195. 10.1186/s13071-018-2773-0 29558995 PMC5859453

[ref35] Marino V Montoya A Mascuñan C : Feline thelaziosis ( *Thelazia callipaeda*) in Spain: state-of-the-art and first prophylactic trial in cats. *J Feline Med Surg.* 2021;23(12):1117–1128. 10.1177/1098612X21997625 33719674 PMC10812156

[ref36] Martínez-Sánchez MI Bolívar-de-Miguel G Cuadros-González J : Ocular thelaziosis: a case report of an emerging zoonosis. *Am J Ophthalmol Case Rep.* 2021;22:101045. 10.1016/j.ajoc.2021.101045 33718660 PMC7933709

[ref37] Masny A Gołąb E Cielecka D : Vector-borne helminths of dogs and humans - focus on central and eastern parts of Europe. *Parasit Vectors.* 2013;6:38. 10.1186/1756-3305-6-38 23433039 PMC3606372

[ref38] Merkel D : Docker: lightweight Linux containers for consistent development and deployment. *Linux J.* 2014;2014,239:2. Reference Source

[ref39] Miró G Montoya A Hernández L : *Thelazia callipaeda*: infection in dogs: a new parasite for Spain. *Parasit Vectors.* 2011;4:148. 10.1186/1756-3305-4-148 21791108 PMC3158752

[ref40] Miterpáková M Trbolová A Hurníková Z : *Thelazia callipaeda* in Slovakia - from sporadic cases to endemic areas. *Parasitol Int.* 2022;87:102495. 10.1016/j.parint.2021.102495 34737070

[ref41] Morgado ACT Vale B do Ribeiro P : First report of human *Thelazia callipaeda* infection in Portugal. *Acta Trop.* 2022;231:106436. 10.1016/j.actatropica.2022.106436 35364047

[ref42] Motta B Nägeli F Nägeli C : Epidemiology of the eye worm *Thelazia callipaeda* in cats from southern Switzerland. *Vet Parasitol.* 2014;203(3–4):287–293. 10.1016/j.vetpar.2014.04.009 24810375

[ref43] Otranto D Cantacessi C Testini G : *Phortica variegata* as an intermediate host of *Thelazia callipaeda* under natural conditions: evidence for pathogen transmission by a male arthropod vector. *Int J Parasitol.* 2006;36(10–11):1167–1173. 10.1016/j.ijpara.2006.06.006 16842795

[ref44] Otranto D Dantas-Torres F Mallia E : *Thelazia callipaeda* (Spirurida, Thelaziidae) in wild animals: report of new host species and ecological implications. *Vet Parasitol.* 2009;166(3–4):262–267. 10.1016/j.vetpar.2009.08.027 19782474

[ref45] Otranto D Dutto M : Human thelaziasis, Europe. *Emerg Infect Dis.* 2008;14(4):647–649. 10.3201/eid1404.071205 18394285 PMC2570937

[ref46] Otranto D Ferroglio E Lia RP : Current status and epidemiological observation of *Thelazia callipaeda* (Spirurida, Thelaziidae) in dogs, cats and foxes in Italy: a “coincidence” or a parasitic disease of the Old Continent? *Vet Parasitol.* 2003;116(4):315–325. 10.1016/j.vetpar.2003.07.022 14580802

[ref47] Otranto D Lia RP Buono V : Biology of *Thelazia callipaeda* (Spirurida, Thelaziidae) eyeworms in naturally infected definitive hosts. *Parasitology.* 2004;129(Pt 5):627–633. 10.1017/s0031182004006018 15552407

[ref48] Otranto D Lia RP Cantacessi C : Nematode biology and larval development of *Thelazia callipaeda* (Spirurida, Thelaziidae) in the drosophilid intermediate host in Europe and China. *Parasitology.* 2005;131(Pt 6):847–855. 10.1017/S0031182005008395 16336738

[ref49] Otranto D Mendoza-Roldan JA : Dantas-Torres F: *Thelazia callipaeda.* *Trends Parasitol.* 2021;37(3):263–264. 10.1016/j.pt.2020.04.013 32451294

[ref50] Otranto D Traversa D : *Thelazia* eyeworm: an original endo- and ecto-parasitic nematode. *Trends Parasitol.* 2005;21(1):1–4. 10.1016/j.pt.2004.10.008 15639731

[ref51] Papadopoulos E Komnenou A Karamanlidis AA : Zoonotic *Thelazia callipaeda* eyeworm in brown bears ( *Ursus arctos*): a new host record in Europe. *Transbound Emerg Dis.* 2022;69(2):235–239. 10.1111/tbed.14414 34889529

[ref52] Papadopoulos E Komnenou A Thomas A : Spreading of *Thelazia callipaeda* in Greece. *Transbound Emerg Dis.* 2018;65(1):248–252. 10.1111/tbed.12626 28239956

[ref53] Paradžik MT Samardžić K Živičnjak T : *Thelazia callipaeda* - first human case of thelaziosis in Croatia. *Wien Klin Wochenschr.* 2016;128(5–6):221–223. 10.1007/s00508-015-0889-1 26542130

[ref54] Pimenta P Cardoso L Pereira MJ : Canine ocular thelaziosis caused by *Thelazia callipaeda* in Portugal. *Vet Ophthalmol.* 2013;16(4):312–315. 10.1111/j.1463-5224.2012.01074.x 23067512

[ref55] Ranallo-Benavidez TR Jaron KS Schatz MC : Genome Scope 2.0 and Smudgeplot for reference-free profiling of polyploid genomes. *Nat Commun.* 2020;11(1):1432. 10.1038/s41467-020-14998-3 32188846 PMC7080791

[ref56] Rao SSP Huntley MH Durand NC : A 3D map of the human genome at kilobase resolution reveals principles of chromatin looping. *Cell.* 2014;159(7):1665–1680. 10.1016/j.cell.2014.11.021 25497547 PMC5635824

[ref57] Rhie A McCarthy SA Fedrigo O : Towards complete and error-free genome assemblies of all vertebrate species. *Nature.* 2021;592(7856):737–746. 10.1038/s41586-021-03451-0 33911273 PMC8081667

[ref58] Rhie A Walenz BP Koren S : Merqury: reference-free quality, completeness, and phasing assessment for genome assemblies. *Genome Biol.* 2020;21(1):245. 10.1186/s13059-020-02134-9 32928274 PMC7488777

[ref59] Rodrigues FT Cardoso L Coutinho T : Ocular thelaziosis due to *Thelazia callipaeda* in a cat from northeastern Portugal. *J Feline Med Surg.* 2012;14(12):952–954. 10.1177/1098612X12459645 22968199 PMC11108008

[ref60] Sargo R Loureiro F Catarino AL : First report of *Thelazia callipaeda* in red foxes ( *Vulpes vulpes*) from Portugal. *J Zoo Wildl Med.* 2014;45(2):458–460. 10.1638/2013-0294R.1 25000721

[ref61] Silva LMR Spoerel S Wiesner L : Ophthalmic *Thelazia callipaeda* infections: first feline and new canine imported cases in Germany. *Parasitol Res.* 2020;119(9):3099–3104. 10.1007/s00436-020-06785-2 32627079 PMC7431390

[ref62] Soares C Sousa SR Anastácio S : Feline thelaziosis caused by *Thelazia callipaeda* in Portugal. *Vet Parasitol.* 2013;196(3–4):528–531. 10.1016/j.vetpar.2013.03.029 23611041

[ref63] Sobotyk C Dietrich J Verocai GG : *Thelazia callipaeda* eyeworms in American black bear, Pennsylvania, USA, 2023. *Emerg Infect Dis.* 2024;30(9):1961–1964. 10.3201/eid3009.240679 39174020 PMC11346993

[ref64] Tasić-Otašević S Gabrielli S Trenkić-Božinović M : Eyeworm infections in dogs and in a human patient in Serbia: a One Health approach is needed. *Comp Immunol Microbiol Infect Dis.* 2016;45:20–22. 10.1016/j.cimid.2016.01.003 27012916

[ref65] Uliano-Silva M Ferreira JGRN Krasheninnikova K : Mito HiFi: a python pipeline for mitochondrial genome assembly from PacBio high fidelity reads. *BMC Bioinform.* 2023;24(1):288. 10.1186/s12859-023-05385-y 37464285 PMC10354987

[ref66] Vasimuddin M Misra S Li H : Efficient architecture-aware acceleration of BWA-MEM for multicore systems. In: IEEE International Parallel and Distributed Processing Symposium (IPDPS) *.* IEEE;2019;314–324. 10.1109/IPDPS.2019.00041

[ref67] Vieira L Rodrigues FT Costa A : First report of canine ocular thelaziosis by *Thelazia callipaeda* in Portugal. *Parasit Vectors.* 2012;5:124. 10.1186/1756-3305-5-124 22720837 PMC3431252

[ref68] Zhou C McCarthy SA Durbin R : YaHS: Yet another Hi-C Scaffolding tool. *Bioinformatics.* 2023;39(1):btac 808. 10.1093/bioinformatics/btac808 36525368 PMC9848053

