# Supplementary material for: The genome sequence of a nematode, Thelazia callipaeda Railliet & Henry, 1910 (Rhabditida: Thelaziidae)
Source: Wellcome Open Res. 2026 Aug 3;10:544. Originally published 2025 Oct 3. [Version 2] doi: 10.12688/wellcomeopenres.24942.2 (PMC13560547; doi:10.12688/wellcomeopenres.24942.2)
Supplement: Peer review history

# Reviewer report: Reviewer response for version 2

**DOI:** 10.21956/wellcomeopenres.30150.r163677

**Published:** 2026-08-26

**Authors:** Erich M Schwarz

**Affiliations:** 1 Cornell University, Ithaca, New York, USA

Martins et al. present a new chromosomally-scaffolded reference-quality genome assembly for the parasitic nematode *Thelazia callipaeda*.  Their genome analysis is generally of high quality, and undoubtedly provides a great improvement on the previous *T. callipaeda* genome assembly published by the 50 Helminth Genomes Project.

 I have the following questions and proposed revisions.

 1. In the Background: "Among these, *Thelazia callipaeda*, is a vector-borne nematode..." should read "Among these, *Thelazia callipaeda* is a vector-borne nematode..." (i.e., delete the second comma).

 2. In the "Genome assembly" section of Methods: the authors write "...database of k-mer counts (k = 31) was generated from the filtered [PacBio HiFi] reads..."   However, nowhere in the Methods do they describe how the HiFi reads were filtered.  Please add a description of the HiFi filtering step.

 3a. In the "Assembly curation" section of Methods, this passage:

 "The X chromosome was identified by read coverage."

 should probably instead read:

 "PacBio reads were aligned to the assembly using minimap2 (Li, 2018) and processed with SAMtools (Danecek et al., 2021) to generate coverage tracks. The X chromosome was identified by read coverage."

 3b. In the "Assembly analysis and quality assessment" section, this passage:

 "PacBio reads were aligned to the assembly using minimap2 (Li, 2018) and processed with SAMtools (Danecek et al., 2021) to generate coverage tracks. It runs BUSCO (Manni et al., 2021) using lineages identified from NCBI Taxonomy."

 should probably instead read:

 "It runs BUSCO (Manni et al., 2021) using lineages identified from NCBI Taxonomy."

 3c. In other words, I think the sentence about aligning PacBio reads with minimap2 in the "Assembly analysis and quality assessment" section is misplaced, and should in fact have been placed in the "Assembly curation" section immediately ahead of the text about identifying the X chromosome through its read coverage.  If the authors agree with me about this, then please implement these revisions.   If the authors do not agree with me about this, then please consider the confusing nature of the existing text and make *some* appropriate revisions.

 4. In Figure 3, the sequence identities listed on the right column:

 "OZ239430.1"

 "OZ239431.1"

 "OZ239432.1"

 "OZ239433.1"

 might be clearer to the reader if they had their simple, human-readable chromosome names appended to them in parentheses, as follows:

 "OZ239430.1

         (1)"

 "OZ239431.1

         (2)"

 "OZ239432.1

         (3)"

 "OZ239433.1

         (X)"

 5. In the "Assembly statistics" section of the Genome Sequence Report, the authors write:

 "The unplaced scaffolds may represent fragments of a highly degenerate Y chromosome, as the HiFi data originated from a mated female (containing male-derived reads). It is also possible that they represent B chromosome fragments."

 This is interesting, but it is presented to the reader without any literature references that would lead to an explanation of the biological reasoning here.  It might be better if both of these sentences cited an appropriate recent reference dealing with Y chromosomes in clade III nematodes (which are biologically nontrivial, because many nematodes instead have an XO/XX sex chromosomal system) and with B chromosomes (a topic that even many reasonably knowledgeable nematode genome analysts may not be familiar).  For instance, the two sentences might be revised as follows:

 "The unplaced scaffolds may represent fragments of a highly degenerate Y chromosome ([add this citation:] Refer (1), as the HiFi data originated from a mated female (containing male-derived reads). It is also possible that they represent B chromosome fragments ([add this citation:] Refer (2).

Are sufficient details of methods and materials provided to allow replication by others?

Yes

Is the rationale for creating the dataset(s) clearly described?

Yes

Are the datasets clearly presented in a useable and accessible format?

Yes

Are the protocols appropriate and is the work technically sound?

Yes

Reviewer Expertise:

Comparative nematode genomics, molecular biology/genetics of nematodes, and parasitology focused on parasitic nematodes.

I confirm that I have read this submission and believe that I have an appropriate level of expertise to confirm that it is of an acceptable scientific standard.

## References

[1] : The genome of Litomosoides sigmodontis illuminates the origins of Y chromosomes in filarial nematodes. *PLOS Genetics* .2024;20(1) : 10.1371/journal.pgen.1011116 10.1371/journal.pgen.1011116 PMC1081718538227589

[2] : Properties and Prospects of B Chromosomes. *Annual Review of Genetics* .2026; 10.1146/annurev-genet-013026-085256 10.1146/annurev-genet-013026-085256 42378843

# Reviewer report: Reviewer response for version 2

**DOI:** 10.21956/wellcomeopenres.30150.r163115

**Published:** 2026-08-20

**Authors:** Jennifer McIntyre

**Affiliations:** 1 University of Glasgow, Glasgow, England, UK

Thank you for the updates.

Are sufficient details of methods and materials provided to allow replication by others?

Yes

Is the rationale for creating the dataset(s) clearly described?

Yes

Are the datasets clearly presented in a useable and accessible format?

Yes

Are the protocols appropriate and is the work technically sound?

Yes

Reviewer Expertise:

I am a veterinary parasitologist. Related to this article I have had experience manually curating a genome assembly for a nematode Teladorsagia circumcincta.

I confirm that I have read this submission and believe that I have an appropriate level of expertise to confirm that it is of an acceptable scientific standard.

# Reviewer report: Reviewer response for version 1

**DOI:** 10.21956/wellcomeopenres.27474.r135283

**Published:** 2025-11-03

**Authors:** Jennifer McIntyre

**Affiliations:** 1 University of Glasgow, Glasgow, England, UK

**Summary of Article**

 The data note describes the production of a chromosomal-level reference assembly for *Thelazia callipaeda*, a vector transmitted zoonotic eye worm. It was made from a single adult female collected in Portugal, with a second specimen used for Hi-C. The genome quality is high, with four chromosomes, one of which is a sex chromosome. In addition, a number of unplaced, but chromosome associated scaffolds are present, as well as entirely unplaced scaffolds, which the authors suggest may represent either a degenerate Y chromosome (impregnated female used) or B chromosomes. From the plot it looks like these unplaced scaffolds did not identify as 'nematoda' using the BlobToolKit pipeline, which could indicate missing data in the database or non-nematode sequence. The genome assembly (both nuclear and mitochondrial) has been made available. It is a significant improvement on a previous assembly. 

**Please address the following points:**

 The authors report that an adult female was used for the PacBio HiFi, but do not then specify what lifestage was used for the Hi-C data. Also, they do not specify what host(s) these were collected from. What animal were the worms collected from? This could be of interest in the future for anyone researching host adaptation, or for understanding any possible contaminants in the sequencing data if present. Was the Hi-C worm collected from the same host as that for the PacBio? What lifestage and sex was it? This could be of interest particularly given that you are wondering whether the unplaced scaffolds could be degenerate Y chromosome.

 The sample details of the genome assemblies and sample data says the sex was 'not collected' yet the paper says it was an adult female for the PacBio. Is this possible to be changed for those accessing the data without first finding the article in the future?

**Other/Minor typos which could be changed if not auto-generated:**

 - Thermomixer not Thermomix?

 - This does not make sense as written: "Normalised libraries were quantified again and equimolar and/or weighted 2.8 nM pools"

 - "The pipeline aligns PacBio reads using minimap2  to generate coverage tracks." I think you mean that Samtools generates the coverage tracks - it has no role in alignment.

 - Reference typo? (schoch2020NCBITaxonomy?)

 - The 'NT database' is the NCBI one, correct?

Are sufficient details of methods and materials provided to allow replication by others?

Yes

Is the rationale for creating the dataset(s) clearly described?

Yes

Are the datasets clearly presented in a useable and accessible format?

Yes

Are the protocols appropriate and is the work technically sound?

Yes

Reviewer Expertise:

I am a veterinary parasitologist. Related to this article I have had experience manually curating a genome assembly for a nematode Teladorsagia circumcincta.

I confirm that I have read this submission and believe that I have an appropriate level of expertise to confirm that it is of an acceptable scientific standard, however I have significant reservations, as outlined above.

# Reviewer report: Reviewer response for version 1

**DOI:** 10.21956/wellcomeopenres.27474.r135280

**Published:** 2025-10-27

**Authors:** Anna Karnkowska

**Affiliations:** 1 Institute of Evolutionary Biology, Biological and Chemical Research Centre, Faculty of Biology, University of Warsaw, Warsaw, Warsaw, Poland

Thelazia callipaeda, the oriental eyeworm, is a parasitic nematode that infects humans and animals via flies (Phortica variegata), causing the zoonotic disease thelaziasis. The presented genome assembly provides valuable insights into its biology and phylogeny, and may help in identifying potential drug targets. The work is clearly presented, and all protocols are state of the art. I have only minor comments regarding the results.

 Approximately 24% of the genome is unplaced and not included in the main chromosomes. These contigs require further investigation to determine which genes they contain and whether they have functional significance.

 BUSCO completeness is approximately 92%, which is somewhat lower than expected given other genome quality metrics. It is important to identify which genes are missing and whether this reflects known gaps in nematode gene content or limitations of the assembly.

 It would also be valuable to examine the distribution of BUSCO genes across contigs to determine whether any are located on unplaced contigs, which could indicate essential genes outside the main chromosomes.

Are sufficient details of methods and materials provided to allow replication by others?

Yes

Is the rationale for creating the dataset(s) clearly described?

Yes

Are the datasets clearly presented in a useable and accessible format?

Yes

Are the protocols appropriate and is the work technically sound?

Yes

Reviewer Expertise:

genomics, microbial eukaryotes, evolutionary biology, symbiosis

I confirm that I have read this submission and believe that I have an appropriate level of expertise to confirm that it is of an acceptable scientific standard.
